# 402. Zoster Vaccination in People Living with HIV Is Associated with Reduced Mortality and Cardiovascular Risk: A Real-World Matched Cohort Study

**DOI:** 10.1093/ofid/ofaf695.140

**Published:** 2026-01-11

**Authors:** Ali Dehghani, George Yendewa

**Affiliations:** Department of Medicine, Case Western Reserve University School of Medicine, Cleveland, OH; Department of Medicine, Case Western Reserve University School of Medicine, Cleveland, OH

## Abstract

**Background:**

People living with HIV (PLWH) face elevated risk of adverse cardiovascular and neurodegenerative outcomes due to persistent immune activation. Herpes zoster (HZ), common in PLWH, may further potentiate these risks. We assessed whether prior zoster vaccination reduces major adverse cardiovascular events (MACE)—a composite of sudden cardiac death, stroke, myocardial infarction, and pulmonary embolism, as well as dementia, all-cause mortality, and psychiatric morbidity (anxiety, depression and schizophrenia) in PLWH without prior HZ.

**Figure:**
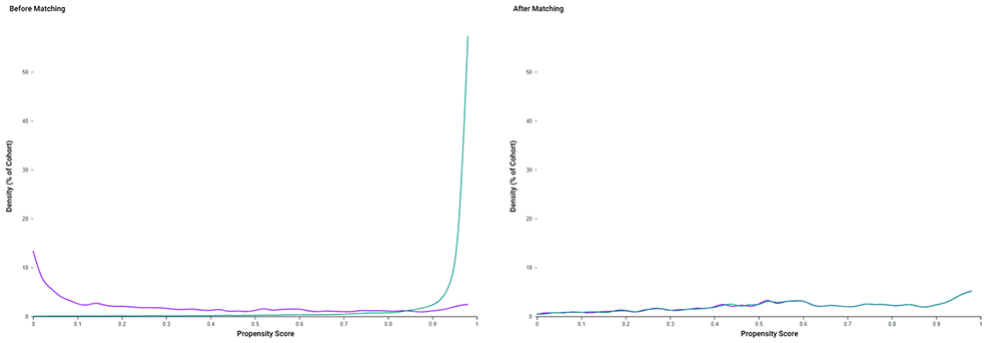
Propensity Score Distribution Before and After Matching (Zoster Vaccinated vs. Unvaccinated PLWH) Density plots of propensity scores for people living with HIV (PLWH) who received zoster vaccination (purple) and those unvaccinated (teal), shown before (left) and after (right) 1:1 matching. Prior to matching, there was substantial imbalance between cohorts, with vaccinated individuals having lower baseline propensity scores. After matching on demographics, ART regimen, comorbidities, psychiatric history, and vaccine exposures, the distributions aligned closely, indicating adequate covariate balance.

**Figure:**
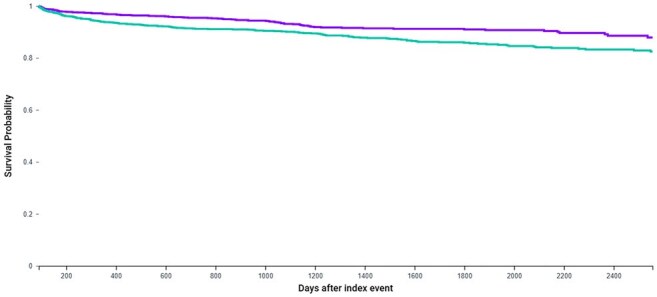
Kaplan-Meier Survival Curve for Major Adverse Cardiovascular Events (MACE) in PLWH by Zoster Vaccination Status Kaplan-Meier analysis comparing time to MACE in people living with HIV (PLWH) who received prior zoster vaccination (purple) versus those unvaccinated (teal), over a follow-up period of 90 days to 7 years post-index. Vaccinated individuals exhibited higher MACE-free survival (HR 0.614, 95% CI: 0.481–0.783; p = 0.0002), suggesting a potential protective cardiovascular effect of recombinant zoster vaccine in this high-risk population.

**Methods:**

We conducted a retrospective matched cohort study using the TriNetX Analytics Network. Adults aged ≥50 years with HIV and no prior HZ diagnosis were stratified by zoster vaccination status. Exclusions included prior MACE, dementia, CNS infections, ESRD, or recent immunosuppressive therapy. Vaccinated individuals were matched 1:1 to unvaccinated controls on demographics, ART regimen, cardiometabolic and psychiatric history, statin and antihypertensive use, and prior vaccine exposures. Primary outcomes were MACE, dementia, and all-cause mortality; secondary outcomes included psychiatric morbidity, and Parkinsonism. Hazard ratios (HRs) and Kaplan-Meier analyses were used to assess outcome differences, with p < 0.05 considered statistically significant.

**Figure:**
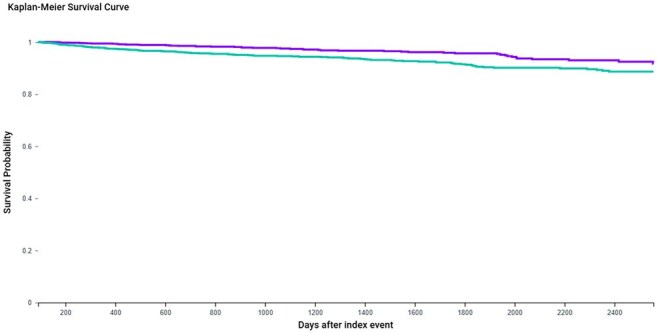
Kaplan-Meier Survival Curve for All-Cause Mortality in PLWH by Zoster Vaccination Status Kaplan-Meier curve illustrating all-cause mortality over 7 years in people living with HIV (PLWH) who received prior zoster vaccination (purple) versus unvaccinated controls (teal). Vaccinated individuals demonstrated significantly improved survival (HR 0.534, 95% CI: 0.380–0.749; p = 0.0002), supporting a protective association between zoster vaccine and long-term mortality in PLWH.

**Figure:**
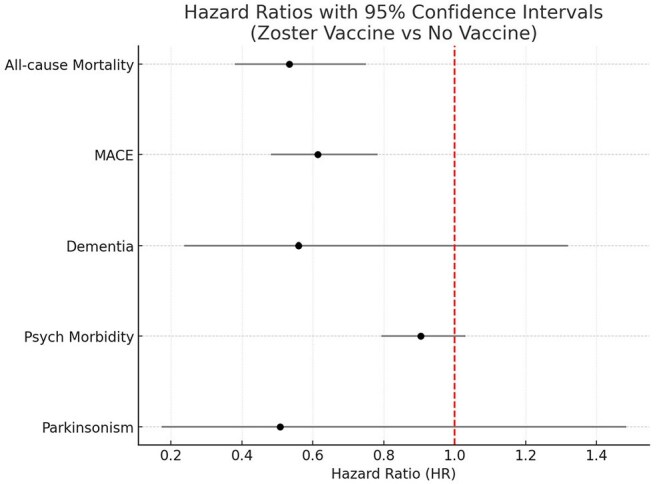
Hazard Ratios for Clinical Outcomes in Zoster-Vaccinated vs. Unvaccinated People Living with HIV (PLWH) Forest plot illustrating hazard ratios (HRs) with 95% confidence intervals (CIs) for major outcomes comparing PLWH who received zoster vaccination prior to herpes zoster infection versus matched unvaccinated controls. Zoster vaccination was associated with significantly reduced hazards of all-cause mortality (HR 0.534, 95% CI: 0.380–0.75), MACE (HR 0.614, 95% CI: 0.481–0.78). A non-significant trend toward lower dementia risk was observed (HR 0.559, 95% CI: 0.237–1.32; p=0.1783). No differences were seen in psychiatric morbidity or Parkinsonism. The red dashed line represents the null value (HR = 1.0).

**Results:**

A total of 3,146 PLWH (50% vaccinated and 50% unvaccinated) were followed from 90 days to 7 years post-index (median follow up: 2.89 years in the vaccinated vs 2.78 years in the unvaccinated group). After matching, the mean age was 58.4 years; 69% were male and 41% were white and 100% were on ART. Zoster vaccination was associated with significantly lower hazards of all-cause mortality (HR 0.534, 95% CI: 0.380–0.749; p=0.0002), MACE (HR 0.614, 95% CI: 0.481–0.783; p=0.0001). Dementia risk trended lower in vaccinated individuals (HR 0.559, 95% CI: 0.237–1.32; p=0.1783) but was not statistically significant. No significant differences were observed in psychiatric morbidity (HR 0.904, 95% CI: 0.793–1.03; p=0.1297) or Parkinsonism (HR 0.507, 95% CI: 0.173–1.483; p=0.2060).

**Conclusion:**

Zoster vaccination in PLWH was associated with significantly reduced risks of all-cause mortality and MACE. Dementia risk trended lower but was not statistically significant.

**Disclosures:**

All Authors: No reported disclosures

